# Gender-related and geographic trends in interactions between radiotherapy professionals on Twitter

**DOI:** 10.1016/j.phro.2022.11.002

**Published:** 2022-11-09

**Authors:** Thomas Berger, Neree Payan, Emmanuelle Fleury, Angela Davey, Abigail Bryce-Atkinson, Eliana Vasquez Osorio, Zhuolin Yang, Thomas McMullan, Leila E.A. Shelley, Anne Gasnier, Jenny Bertholet, Marianne C. Aznar, William H. Nailon

**Affiliations:** a Department of Oncology Physics, Edinburgh Cancer Centre, Western General Hospital, Crewe Road South, Edinburgh, Scotland, UK; b Patrick G Johnston Centre for Cancer Research, Queen’s University Belfast, Belfast, UK; c Department of Radiation Oncology, Erasmus Medical Center, Rotterdam, Netherlands; d Department of Radiation Oncology, HollandPTC, Delft, Netherlands; e Division of Cancer Sciences, Faculty of Biology, Medicine and Health, The University of Manchester, Manchester, England, UK; f School of Engineering, the University of Edinburgh, the King’s Buildings, Mayfield Road, Edinburgh, Scotland, UK; g Radiotherapy Department, Gustave Roussy Cancer Campus, Villejuif, France; h Division of Medical Radiation Physics and Department of Radiation Oncology, Inselspital, Bern University Hospital and University of Bern, Bern, Switzerland

**Keywords:** Radiotherapy, ESTRO, ESTRO congress, Virtual congress, Social media, Gender bias, Trends, Twitter, COVID-19

## Abstract

**Background and purpose:**

Twitter presence in academia has been linked to greater research impact which influences career progression. The purpose of this study was to analyse Twitter activity of the radiotherapy community around ESTRO congresses with a focus on gender-related and geographic trends.

**Materials and methods:**

Tweets, re-tweets and replies, here designated as *interactions*, around the ESTRO congresses held in 2012–2021 were collected. Twitter activity was analysed temporally and, for the period 2016–2021, the geographical span of the ESTRO Twitter network was studied. Tweets and Twitter users collated during the 10 years analysed were ranked based on number of ‘likes’, ‘re-tweets’ and followers, considered as indicators of leadership/influence. Gender representation was assessed for the top-end percentiles.

**Results:**

Twitter activity around ESTRO congresses was multiplied by 60 in 6 years growing from 150 interactions in 2012 to a peak of 9097 in 2018. In 2020, during the SARS-CoV-2 pandemic, activity dropped by 60 % to reach 2945 interactions and recovered to half the pre-pandemic level in 2021. Europe, North America and Oceania were strongly connected and remained the main contributors. While overall, 58 % of accounts were owned by men, this proportion increased towards top liked/re-tweeted tweets and most-followed profiles to reach up to 84 % in the top-percentiles.

**Conclusion:**

During the SARS-CoV-2 pandemic, Twitter activity around ESTRO congresses substantially decreased. Men were over-represented on the platform and in most popular tweets and influential accounts. Given the increasing importance of social media presence in academia the gender-based biases observed may help in understanding the gender gap in career progression.

## 1 Introduction

High-profile social movements have recently put the focus in our societies on gender inequities. The momentum generated incited institutions and individuals to reflect on these questions, including in the academic community. A growing body of evidence now indicates that gender-based biases influence, sometimes unconsciously, the healthcare work environment and the inter-personal relationships within it [1]. Specifically, women in academic medicine, medical oncology and radiation oncology have made steady gains in recent decades but remain underrepresented in the upper echelons and still face persistent career inequities [2], [3]. Previous studies also highlighted the gender pay gap favouring male healthcare professionals and researchers [4], [5], [6]. However, it is worth noting that there are radiotherapy initiatives that aim at mitigating these disparities in the field by providing networking and mentoring opportunities for women.

In recent decades, the field of radiotherapy has undergone profound evolutions largely driven by the digital transformation of society [7]. In the last 10 years, the role of social media has become increasingly important with more than a third of humanity using Facebook monthly [8]. This also holds true for healthcare professionals, with a global radiotherapy Twitter network found to spread from 23 to 116 countries between 2014 and 2019 [9], [10]. All the interactions undertaken on social media leave a digital mark which allows for quantitative and systematic investigation. For example, a previous study found that Twitter users discussing radiotherapy topics were predominantly healthcare professionals [11]. These aspects make Twitter a particularly useful resource for learning more, in a quantitative way, about the wider radiotherapy community, its geographical and gender distribution and the way its members interact. Twitter presence in academia was also linked to greater research impact [12], [13], [14], [15], [16], [17] which plays a role in career progression. Given the increasing importance of social media in academia, it is important to assess its role on gender-based disparities as a possible inhibitor or contributor to equal opportunity.

During international radiotherapy congresses community members connect with one another and disseminate their research, which may result in increased activity on Twitter. One such event is the annual congress of the European Society for Radiotherapy and Oncology (ESTRO). In 2020 the COVID-19 pandemic compelled the ESTRO society to cancel the plenary setting of the congress and to hold it in virtual format. This may have driven more radiotherapy professionals to social media platforms to compensate for the lack of in-person interactions. The change to virtual platforms has had profound implications on the demographics of conference attendance across different fields, in particular increased attendance by women [18].

The purpose of this study was to analyse the activity on Twitter around ESTRO congresses to reveal salient characteristics of the ESTRO community interacting on Twitter, with a focus on gender-related and geographic trends.

## 2 Materials and methods

### 2.1 Temporal and geographic evolution of Twitter use

Posts on Twitter (tweets), re-tweets^1^^†^ and replies to tweets related to ESTRO were retrieved for the period covering the past ten ESTRO congresses (from 2012 to 2021). For the remainder of this article, the term *interaction* refers to interpersonal communications (re-tweets, replies) as well as original tweets.

The Twitter application programming interface was queried to retrieve activity dealing with ESTRO that originated around each of the annual congress ±5 days. For example, the keywords used for the year 2021 were #ESTRO, #ESTRO2021 and #ESTRO40. The full list of keywords is detailed, in addition to congresses’ locations and dates, in Supplementary Material A.

The temporal evolution of the number of collected Twitter interactions was analysed. For the period 2016 to 2021 where the total number of communications grew above 1000, the connections that took place between persons re-tweeting or replying to an original tweet, and the author of the tweet in question were mapped, using the users’ locations, to analyse the evolution of the geographical network of collaborators.

### 2.2 Gender-related trends and influence on Twitter

Communications were studied on a sub-sample of the data excluding Twitter accounts that represented associations, corporations or individuals using the #ESTRO for opportunistic purposes (such as promoting products, advertising, pornography etc…). This excluded 1463 of the 4258 accounts from further analysis. The pronouns listed in a user’s profile description were used to categorise gender into two categories (male, female) with a caveat that this oversimplified description does not include non-binary, gender-fluid, or gender-neutral individuals. For individuals who did not have pronouns listed, information provided on a users’ profile (name, picture, description) was used to assign gender. If gender could not be assigned from this information the individual was classified as unidentified.

Liking or re-tweeting someone else’s tweet gives more audience and credibility to the message and the originator. These actions can also be interpreted, to some level, as expressing support, endorsement or even a sign of deference. Therefore, the number of ‘likes’, re-tweets and followers were used as quantitative indicators of popularity, leadership/authority and influence.

To determine whether tweets authored by individuals of a specific gender trigger more such actions from the community, all tweets retrieved were pooled and ranked by number of 1) likes and 2) re-tweets, ordered in quartiles and percentiles. The proportion of male and female authors was evaluated for the fourth quartile, the 90th, 95th and 99th percentiles, comprising the top liked/retweeted tweets. The female/male ratio (F/M) in each group was then compared to the one of the whole sample. This analysis was complemented by an individual-specific approach in which Twitter users that interacted more than once were ranked based on the cumulated number of 1) likes, 2) re-tweets and 3) replies to tweets they authored during the past ten congresses and 4) followers. Then, similarly to the approach described above, the F/M ratio was determined for the subgroups of Twitter profiles and compared to the one of the sample. To test for statistical significance of gender imbalance in the percentiles of interest, comparison with gender ratio of the whole sample analysed was performed using Fisher’s exact test.

### 2.3 Gender representation and profession of Twitter users

Gender representation as well as the average number of interactions on Twitter over the ten years studied were assessed for Twitter users who were grouped by professional qualifications. The profiles were classified, when relevant, using the following categories: PhD students, post-doctoral researchers, PhD holders, associate/assistant professors, professors, radiotherapists/radiographers/radiation therapists (RTT), physicists/clinical scientists or clinicians. This categorisation was performed based on the presence of specific keywords shown in Supplementary Material B, in the username, Twitter handle or description.

The data extraction and subsequent analysis were performed using Matlab software (v.R2021b).

## 3 Results

### 3.1 Temporal and geographic evolution of Twitter use

**Fig. 1:**
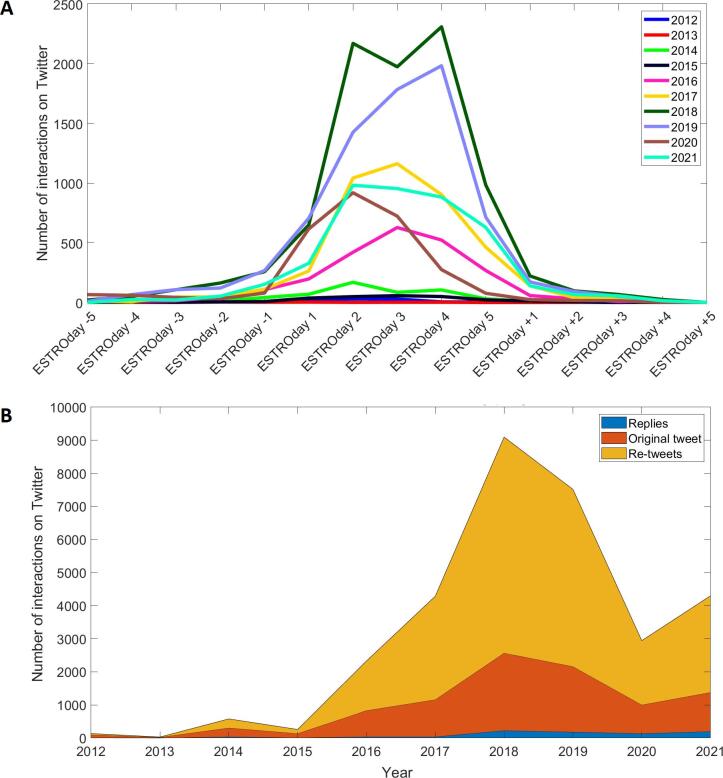
Panel A shows the activity on Twitter during and around (±5 days) the past ten ESTRO congresses. Panel B shows the yearly variation of Twitter activity during ESTRO congresses detailing number of tweets, re-tweets and replies.

Twitter activity related to ESTRO peaked during congresses as shown in Fig. 1A. From 2012 to 2018 the number of interactions substantially increased, going from nearly 150 to a peak of 9097 in 2018 (Fig. 1B). This number decreased in 2019, and dropped to 2945 in 2020, the first year of the SARS-CoV-2 pandemic. It then increased to nearly half the pre-pandemic figures the next year (4302).

**Fig. 3:**
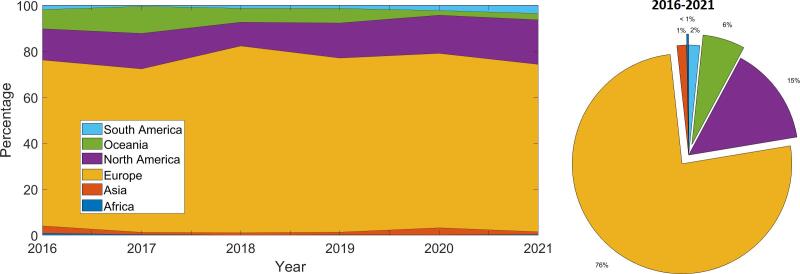
Continent of origin of Twitter communications (Users re-tweeting or replying to tweets and authors of the tweets in question), shown in percentage for the time-interval studied.

The evolution over time of the geographical distribution of the network of collaborators exchanging on Twitter around ESTRO congresses is displayed in the animated map in Fig. 2. Overall, Europe, North America and Oceania were strongly connected and the main contributors accounting for 76 %, 15 % and 6 % of exchanges, respectively (Fig. 3). In addition, contributions from South America progressively increased from 2 % to 4 % during the period studied.

Fig. 2Evolution over time of the geographical distribution of the network of collaborators that communicated via re-tweets or replies to tweets around ESTRO congresses, displayed in video format.
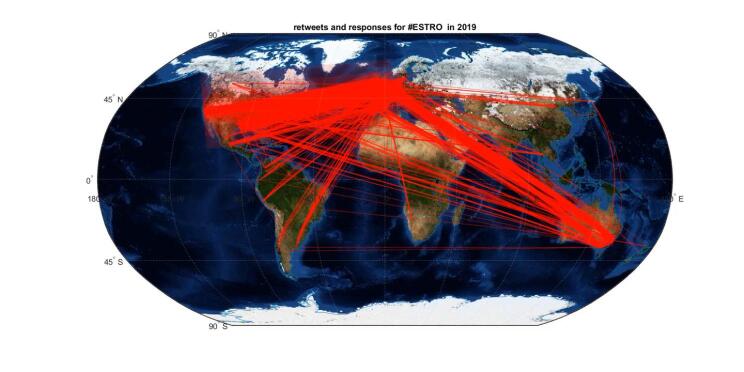

### 3.2 Gender-related trends and influence on Twitter

The 2795 accounts selected were categorised as: 1526 (55 %) male, 1100 (39 %) female and 169 (6 %) profiles with unidentified gender. Excluding those unidentified, gender representation was 58 % male and 42 % female users. This proportion was relatively stable over time, with male/female proportions oscillating as shown in Supplementary Material C.

**Fig. 4:**
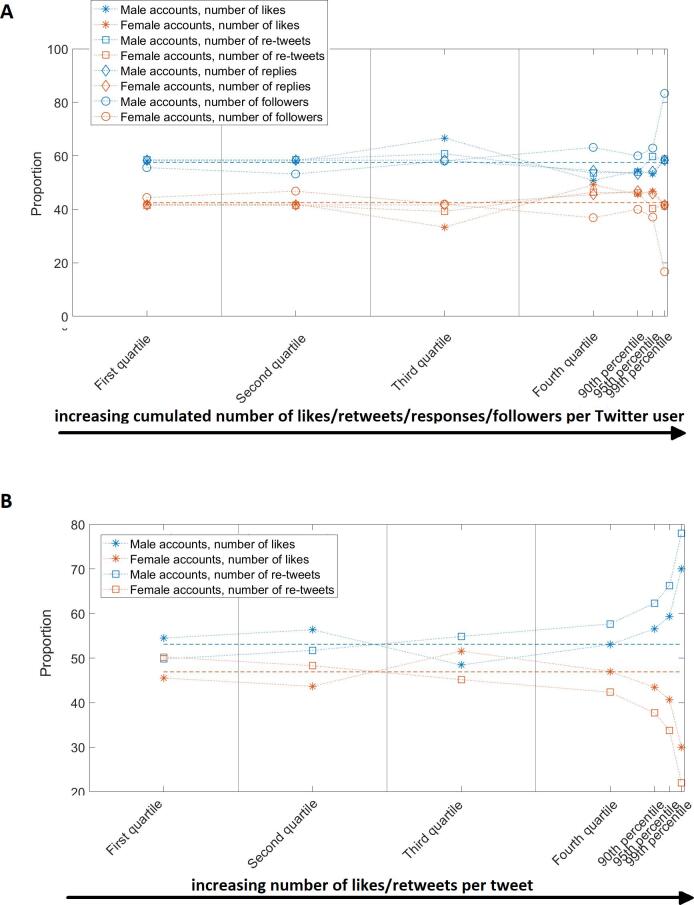
Panel A shows the gender balance of quartiles and percentiles of Twitter users ranked by number of followers as well as number of likes, re-tweets and replies cumulated during the past ten congresses. Panel B shows the proportion of tweets, categorized by gender of the author, ranked from the least-to-most liked and re-tweeted tweets. This second panel presents results from a tweet-specific approach as opposed to an individual-specific approach for the first panel. The dashed lines represent the gender balance of the sample studied and are used as a reference to calculate gender over/under-representation in a given group. Dotted lines are solely used to facilitate the visualisation of trends.

As illustrated in Fig. 4A, women were over-represented in Twitter accounts that cumulated the highest number of likes, re-tweets and replies (top 25 % to top 5 %) for the tweets they authored during the ten congresses analysed. Specifically, there were respectively 49 %, 46 % and 46 % of women in the quartile that cumulated the highest number of likes, re-tweets and replies while women represented 43 % of the sample studied. This corresponds to an over-representation of women ranging from 3 % for number of re-tweets and replies (p greater than 0.20) to 6 % for number of likes (p = 0.04). The trend was opposite for the most followed accounts, with men being over-represented by 6 %, 3 % and 5 % respectively in the top 25 %, 10 % and 5 % most-followed accounts. For the 1 % most followed accounts, this proportion imbalance reached 26 % (p = 0.04).

In the analysis of the impact of tweets (Fig. 4B), those authored by men were over-represented by 0–5 %, 4–9 % and 6–13 % for the top liked and re-tweeted 25 %, 10 % and 5 % respectively, when compared to the gender balance of the sample studied. The proportion of male profiles in accounts that authored the 1 % of tweets that were most liked and re-tweeted exceeded the gender balance of the sample by 17 % (p = 0.02) and 25 % (p < 0.01), respectively.

### 3.3 Gender representation and profession of Twitter users

**Fig. 5:**
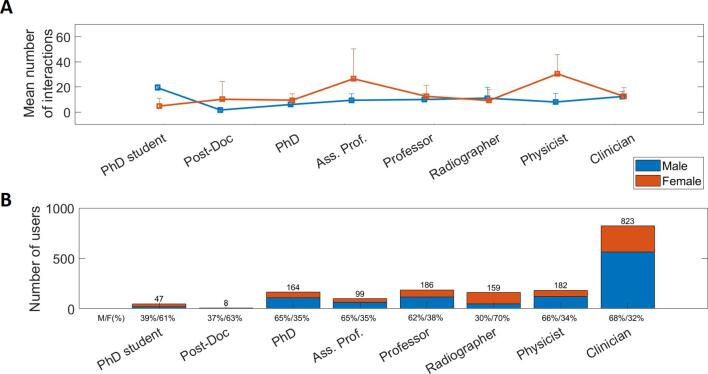
Panel A shows the mean number of cumulated interactions on Twitter per user and over the ten years studied with error bars representing first and third quartiles. Panel B shows the absolute number of accounts with academic titles and professions identified as well as their gender balance. It is important to keep in mind that some categories may overlap. The abbreviation Ass. Prof. comprises both assistant and associate professors and the category radiographers also includes radiotherapists/RTT.

Of the 2626 selected accounts with allocated gender, a professional qualification (radiotherapists/radiographers, physicists/clinical scientists or clinicians) was identified in 1162 of them. These were composed in majority (71 %) by clinicians, with 822 profiles (Fig. 5). Physicists or equivalent, as defined by the keywords presented in Supplementary Material B, was the second group most present on Twitter with 181 accounts (16 %) and radiotherapists/radiographers accounted for 159 accounts (14 %). Academic titles were identified for 504 users who claimed to be: 9 % of PhD students, 2 % post-doctoral researchers, 33 % PhD holders, 20 % ass. professors and 37 % professors. PhD students, post-doctoral researchers and radiotherapists/radiographers were the three categories studied that counted a majority of women with F/M ratios of 61 %/39 %, 63 %/37 % and 70 %/30 %, respectively. In all other categories men outnumbered women with F/M ratios ranging from 38 %/62 % for professors to 32 %/68 % for clinicians. Also, women were on average more active on Twitter than men. Female physicists were the most active with, on average, a total of 30 interactions over the 10 years studied, which is nearly-four times the number of interactions of their male counterparts. Overall, women tweeted more than men with an average number of original tweets of 2.2 against 1.8.

## 4 Discussion

The analysis of 31,483 interactions covering the last ten ESTRO congresses has shown that Twitter activity dropped substantially during the SARS-CoV-2 outbreak, with a 60 % decrease to 2945 interactions in 2020. Most intercommunications originated from Europe, North America, and Oceania with 76 %, 15 % and 6 % respectively. Also, men appear to be over-represented by 10 % on the platform as, 58 % of the profiles with an identified gender were men and 42 % women while the F/M ratio of European ESTRO members is 52 %/48 %. In addition, men were increasingly over-represented towards top liked/re-tweeted tweets and most followed profiles and by up to 26 % in the top 1 %.

Twitter activity during and around ESTRO congresses considerably increased over the past ten years, going from ∼ 150 interactions in 2012 to a peak at 9097 in 2018. ESTRO annual congresses are regularly held in April or May. However, this pattern was disrupted by the SARS-CoV-2 outbreak as, in 2020, the congress was postponed twice to finally take place fully online in December. In 2021 the congress was held in a hybrid format in August. The virtual congress format eases economic and travel-related obstacles, which was found in a previous study to possibly result in increased participation from women, scientists living with a disability, early-career researchers and researchers from countries with limited resources [18]. Considering these results, the observed decrease in activity on Twitter during the 2020 online ESTRO congress appears counter-intuitive although it remarkably mirrors the evolution of the number of ESTRO participants (Supplementary Material D [19]).

The world regions that mostly contributed to communications on Twitter were Europe, North America and Oceania, which are areas that comprise most of the economically-advanced countries, according to the International Monetary Fund definition [20], with the exception of several Eastern Asian nations. Radiation oncology research activities require radiotherapy capabilities while Twitter use depends upon easy internet access, both of which are not widely available in many developing nations. Adding to that, contributions from South America were found to progressively increase in more recent years, reflecting its increased contribution to worldwide radiotherapy literature [7]. Finally, it is interesting to note that the virtual format of the congress did not result in markedly increased attendance from low- and middle-income countries compared to other nations (Fig. 3).

The gender imbalances observed in this study, are fully meaningful when put into perspective considering the gender ratios in the field. The term over-representation was used when, for a sub-group studied, representatives of a specific gender were present in a proportion higher than that of the whole sample. The analysis of the number of likes, re-tweets and replies per tweet, which can be considered as indicators of popularity, leadership and influence, revealed that men were increasingly over-represented towards top liked/re-tweeted tweets. This over-representation reached a maximum of 25 % for the 1 % top-re-tweeted tweets. Conversely, women were over-represented by up to 6 % in the last quartile of Twitter profiles that cumulated the highest number of likes, re-tweets and replies during the period studied. However women over-representation did not follow a monotonic trend, thus, for higher percentiles, a progressive return to gender balance was observed (Fig. 4A). It is interesting to note that, when analysing number of likes and re-tweets, individual- and tweet-specific approaches yielded diverging results. This discrepancy may be explained by the higher activity of women (Fig. 5A), which resulted in higher numbers of accumulated likes and re-tweets per account but fewer likes and re-tweets per tweet. As of the number of followers, which is widely regarded as a marker of influence, an increasing over-representation of men towards the most followed accounts was observed. In particular, men were over-represented by 3–6 % in the top 25 %-to-5 %. This proportion imbalance reached a maximum for the top 1 % most-followed accounts with an over-representation of men of 26 %. The difference between the trend in women and men over-representation (men following a monotonic trend from the top 25 % to top 1 %), can be visualised by comparing the dotted lines on Fig. 4 between Panel A and Panel B. Finally, it is notable to draw a parallel between the disproportionate representation of men in the most influential profiles and tweets and their over-representation in the highest hierarchical positions in our disciplines [3].

The professional qualifications of Twitter users analysed in this study seem to be relatively representative of the ones of ESTRO members [21] with: 71 % of clinicians on Twitter, against 58 % of clinical and radiation oncologists in ESTRO members, 16 % of physicists (or equivalent) against 21 % of medical physicists, and 14 % of radiotherapists/radiographers, against 12 % of RTTs/radiotherapy nurses. In the present study, women were found to outnumber men in three categories analysed: PhD students, post-doctoral researchers and radiotherapists/radiographers with F/M ratios of 61 %/39 %, 63 %/37 % and 70 %/30 %, respectively. In all other categories the proportion of men exceeded the proportion of women with F/M ratios ranging from 38 %/62 % for professors to 32 %/68 % for clinicians. It is also interesting to compare these numbers with gender balance in the corresponding professions across different nations. In the United States for instance, academic radiation oncology faculty, was found to be composed of 31 % of women in 2019 [3]. In the United Kingdom in 2019, 53 % of NHS clinical oncologists were women [22]. In Australia and New Zealand women make up 28 % of the medical physics workforce and 23 % in AAPM members (predominantly USA) [23], [24]. No figures could be found for radiation therapists or radiotherapy industry professionals. It is also remarkable that only 11 % of accounts with an academic title identified claimed to be either PhD students or post-doctoral researchers while we found 57 % of assistant/associate professors or professors. This seems counter-intuitive as research organisations count more PhD students and postdocs than professors and assistant/associate professors. This divergence may be explained by the fact that the individuals tweeting during ESTRO congresses are not representative of the radiotherapy research workforce. Other potential explanations may be related to cultural aspects or that those occupying the lower echelons are less inclined to specify their titles on Twitter.

The list of keywords used to categorise Twitter users based on their professional qualifications resulted from an iterative filtering process in which Twitter account information was systematically reviewed to ensure that no significant account types were left out from the analysis. The imbalance in keyword numbers for different categories is therefore reflecting the imbalance in professional qualifications claimed by the users. In line with this approach, a category focussing on radiobiologist profiles was considered but had to be omitted due to the insufficient number of corresponding profiles to statistically determine gender balance estimates.

To make this analysis possible, three gender categories were used but for the most part, our study focussed on a binary approach to gender which does not reflect the complexity of gender identification. In addition, Twitter does not provide self-reported gender information and gender was therefore allocated based on preferred pronouns when available or based on the authors’ perceptions otherwise.

Social media presence is becoming increasingly important in academia. It has been linked to greater research impact [12], [13], [14], [15], [16] which plays a role in career progression. Therefore, the gender-based inequities observed in this study, while more nuanced than others, may also contribute to this positive feedback loop that prevents women from progressing equally to men in their career.

In conclusion, Twitter activity around ESTRO congresses was multiplied by 60 in 6 years growing from 150 interactions in 2012 to more than 9000 in 2018. During the SARS-CoV-2 outbreak, activity dropped by 60 % in 2020 and then recovered to only half the pre-pandemic level the next year. Europe, North America and Oceania were strongly connected and remained the main contributors. Men were over-represented overall on the platform as well as in most popular tweets and influential accounts. Because of the increasing importance of social media presence in academia, the gender-based biases observed in this study, may help in understanding the gender gap in career progression.

## 5 Data sharing statement

The data analysed in this study are public information which were retrieved from the Twitter Application Programming Interface (API). The systematic access and analysis of this data were subject to permission that was granted to the authors by Twitter. At this occasion, the authors have committed to solely publish data resulting from statistical analysis hence avoiding publication of identifiable information from Twitter users.

## Declaration of Competing Interest

The authors declare that they have no known competing financial interests or personal relationships that could have appeared to influence the work reported in this paper.

## Footnotes

1 † re-tweet: the action of a Twitter user to share with their own audience the tweet of another Twitter user.

## References

[1] Files J.A., Mayer A.P., Ko M.G., Friedrich P., Jenkins M., Bryan M.J. (2017). Speaker introductions at internal medicine grand
rounds: forms of address reveal gender bias. J Women's Health.

[2] Diana M. Lautenberger VMD. The State of Women in Academic Medicine 2018-2019: exploring pathways to equity. 2020 https://store.aamc.org/downloadable/download/sample/sample_id/330/; 2022 [accessed 22 February 2022].

[3] Chowdhary M., Chowdhary A., Royce T.J., Patel K.R., Chhabra A.M., Jain S. (2020). Women’s representation in leadership positions in
academic medical oncology, radiation oncology, and surgical
oncology programs. JAMA Netw Open.

[4] Seabury S.A., Chandra A., Jena A.B. (2013). Trends in the earnings of male and female health care
professionals in the United States, 1987 to 2010. JAMA Intern Med.

[5] Jagsi R., Griffith K.A., Stewart A., Sambuco D., DeCastro R., Ubel P.A. (2012). Gender differences in the salaries of physician
researchers. JAMA.

[6] Ly D.P., Seabury S.A., Jena A.B. (2016). Differences in incomes of physicians in the United
States by race and sex: observational study. BMJ.

[7] Berger T., Noble D.J., Shelley L.E.A., Hopkins K.I., McLaren D.B., Burnet N.G. (2021). 50 years of radiotherapy research: Evolution, trends
and lessons for the future. Radiother Oncol.

[8] Meta - Facebook Reports Third Quarter 2021 Results n.d. https://investor.fb.com/investor-news/press-release-details/2021/Facebook-Reports-Third-Quarter-2021-Results/default.aspx; 2022 [accessed 22 February 2022].

[9] Prabhu A.V., Beriwal S., Ahmed W., Ayyaswami V., Simcock R., Katz M.S. (2021). #radonc: Growth of the global radiation oncology
Twitter network. Clin Transl Radiat Oncol.

[10] Simcock R., Thomas T.V., Estes C., Filippi A.R., Katz M.A., Pereira I.J. (2020). COVID-19: Global radiation oncology’s targeted
response for pandemic preparedness. Clin Transl Radiat Oncol.

[11] Rahimy E., Sandhu N.K., Giao D.M., Pollom E.L. (2021). #TrendingNow: instagram versus twitter activity among
radiation oncology patients and professionals. Pract Radiat Oncol.

[12] Paradis N., Knoll M.A., Shah C., Lambert C., Delouya G., Bahig H. (2020). Twitter: a platform for dissemination and discussion
of scientific papers in radiation oncology. Am J Clin Oncol.

[13] Vaghjiani N.G., Lal V., Vahidi N., Ebadi A., Carli M., Sima A. (2021). Social media and academic impact: do early tweets
correlate with future citations?. Ear Nose Throat J.

[14] Deshpande N., Crossley J.R., Malekzadeh S. (2022). Association between twitter mentions and academic
citations in otolaryngology literature. Otolaryngol Head Neck Surg.

[15] Özkent Y. (2022). Social media usage to share information in
communication journals: an analysis of social media activity and
article citations. PLoS ONE.

[16] Sudah S., Faccone R.D., Nasra M.H., Constantinescu D., Menendez M.E., Nicholson A. (2022). Twitter mentions influence academic citation count of
shoulder and elbow surgery publications. Cureus.

[17] Muren L.P., Redalen K.R., Thorwarth D. (2022). Five years, 20 volumes and 300 publications of physics
and imaging in radiation oncology. Phys Imaging Radiat Oncol.

[18] Skiles M., Yang E., Reshef O., Muñoz D.R., Cintron D., Lind M.L. (2021). Conference demographics and footprint changed by
virtual platforms. Nat Sustain.

[19] ESTRO - About ESTRO n.d. https://www.estro.org/About/Newsroom/Publications; 2022 [accessed 17 March 2022].

[20] World Economic Outlook Database April 2020 -- WEO Groups and Aggregates Information n.d. https://www.imf.org/external/pubs/ft/weo/2020/01/weodata/groups.htm 2022 [accessed 22 February 2022].

[21] ESTRO Annual Report 2019 n.d. https://user-swndwmf.cld.bz/ESTRO-Annual-Report-2019/10/; 2022 [accessed 17 March 2022].

[22] Narrowing of NHS gender divide but men still the majority in senior roles - NHS Digital n.d. https://digital.nhs.uk/news/2018/narrowing-of-nhs-gender-divide-but-men-still-the-majority-in-senior-roles; 2022 [accessed 22 February 2022].

[23] Covington E.L., Moran J.M., Paradis K.C. (2020). The state of gender diversity in medical
physics. Med Phys.

[24] Crowe S.B., Kairn T. (2016). Women in medical physics: a preliminary analysis of
workforce and research participation in Australia and New
Zealand. Australas Phys Eng Sci Med.

