# Supplementary material for: Gender-related and geographic trends in interactions between radiotherapy professionals on Twitter
Source: Phys Imaging Radiat Oncol. 2022 Nov 9;24:129–35. doi: 10.1016/j.phro.2022.11.002 (PMC9696828; doi:10.1016/j.phro.2022.11.002)

Supplementary Materials

A: Dates, locations and search keywords pertaining to the ESTRO congresses analysed.

B: List of keywords used to identify academic titles and professional qualifications of Twitter users.

C: Proportion of Twitter users by gender over the ten years studied.

D: Evolution of number of ESTRO members, participants to the congress and submitted abstracts (data extracted from ESTRO annual reports).

A: Dates, locations and search keywords pertaining to the ESTRO congresses analysed.

year	Conf. nb	Day start	Month start	Day end	Month end	Location	Keywords
2012	31	9	5	13	5	Barcelona	#ESTRO2012 ; #ESTRO31 ; #ESTRO
2013	32	27	9	1	10	Amsterdam	#ESTRO2013 ; #ESTRO32 ; #ESTRO
2014	33	4	4	8	4	Vienna	#ESTRO2014 ; #ESTRO33 ; #ESTRO
2015	34	24	4	28	4	Barcelona	#ESTRO2015 ; #ESTRO34 ; #ESTRO
2016	35	29	4	3	5	Turin	#ESTRO2016 ; #ESTRO35 ; #ESTRO
2017	36	5	5	9	5	Vienna	#ESTRO2017 ; #ESTRO36 ; #ESTRO
2018	37	20	4	24	4	Barcelona	#ESTRO2018 ; #ESTRO37 ; #ESTRO
2019	38	26	4	30	4	Milan	#ESTRO2019 ; #ESTRO38 ; #ESTRO
2020	39	28	11	1	12	Online	#ESTRO2020 ; #ESTRO39 ; #ESTRO
2021	40	27	8	31	8	Madrid /Online	#ESTRO2021 ; #ESTRO40 ; #ESTRO

B: List of keywords used to identify academic titles and professional qualifications of Twitter users.

All keywords listed were by default case insensitive unless stated otherwise.

Keywords for Medical Doctors category:
In the user's description:
"Medico"
"Radiooncóloga"
"M.D"
"physician"
"chirurgien"
"urologist"
"cancer specialist"
"doctor"
"Oncólogo"
"rad onc"
"radonc"
"oncologist"
"surgeon"
"MD" (case sensitive)
In the user's Twitter handle:
"MD" (case sensitive)
In the username:
"MD" (case sensitive)

Keywords for Physicists or equivalent category:
In the user's description:
"medisinsk fysikk"
"clinical scientist"
"Engineer"
"Física Médica"
"Físico"
"physicist"
In the user's Twitter handle:
"physicist"
In the username:
"physicist"

Keywords for Radiation Therapists or equivalent category:
In the user's description:
"Oncology Clinical Research Nurse"
"nurse (oncology)"
"cancer Nurse"
"oncology Nurse"
"radiographer"
"radiation therapist"
"rtt"
In the user's Twitter handle:

"rtt"
In the username:
"rtt"

Keywords for PhD category:
In the user's description:
"Ph.D"
"phd"
In the user's Twitter handle:
"phd"
In the username:
"phd"
As we wanted PhD students NOT to be included in this category, the presence of the following keywords resulted in the exclusion of the Twitter user:
In the user's description:
"student"
"candidate"

Keywords for Professors:
In the user's description:
"prof" (this keyword being included in 'professor', users using 'professor' in their description are also selected)
In the user's Twitter handle:
"prof"
In the username:
"prof"

As we wanted Associate or Assistant Professors NOT to be included in this category, the presence of the following keywords resulted in the exclusion of the Twitter user:
In the user's description:
"associate"
"assistant"
"asst"
"ass."
"ass prof"

Keywords for Assistant/Associate Professors:
In the user's description:
"Assoc Prof"
"Assoc. Prof"
"Assoc.Prof"
"prof ass"
"Ass prof"
"Assistant prof"
"Ass. prof"

"asst prof"
"associate prof"

Keywords for PostDocs:
In the user's description:
"postdok"
"postdoc"
"post doc"
"post-doc"

Keywords for PhD students:
In the user's description:
"PhDstud"
"PhD stud"
"Ph.D stud"
"PhD cand"
"Ph.D cand"
"PhDcand"
"PhD Fellow"
"Ph.D Fellow"

C: Proportion of Twitter users by gender over the ten years studied.

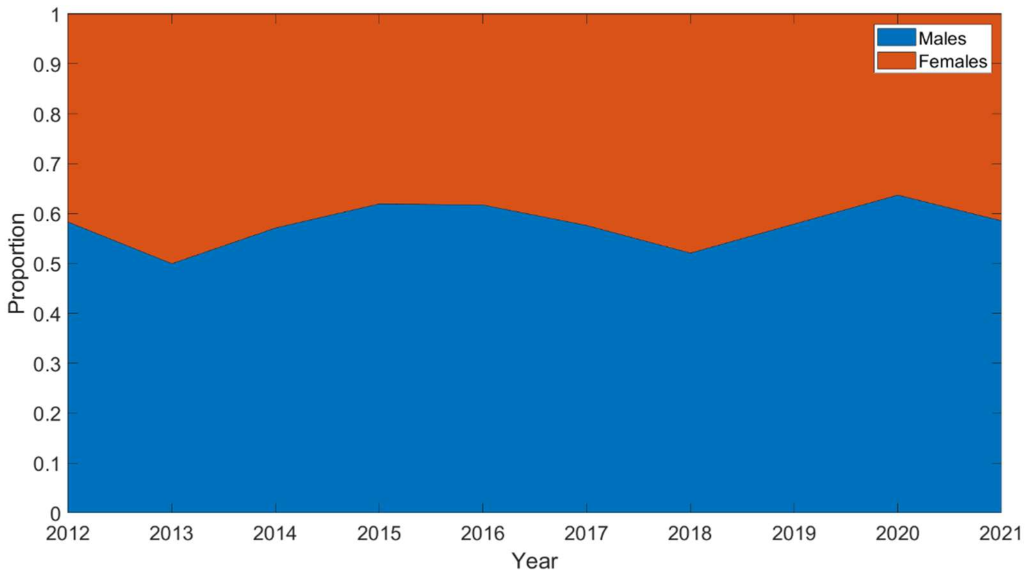

D: Evolution of number of ESTRO members, participants to the congress and submitted abstracts (data extracted from ESTRO annual reports).

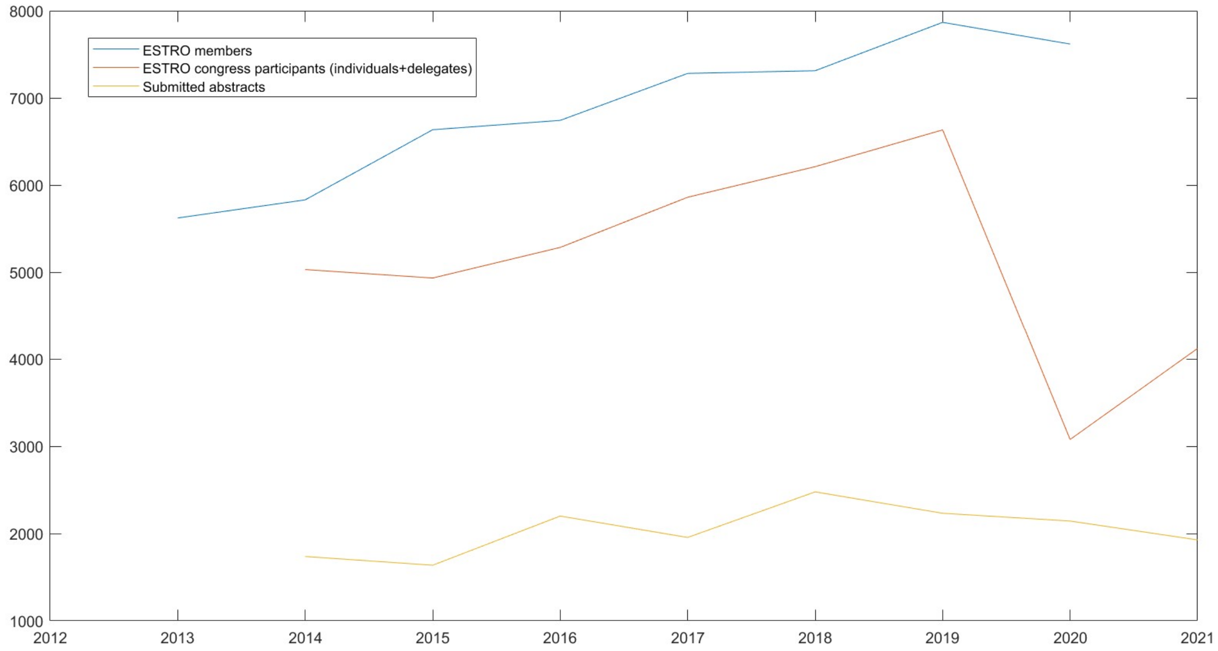

Supplement: Supplementary data 1 [file mmc1.pdf]
